# Evaluation of the Structure and Geometric Properties of Crushed Igneous Rock Aggregates

**DOI:** 10.3390/ma14237202

**Published:** 2021-11-25

**Authors:** Paweł Strzałkowski, Magdalena Duchnowska, Urszula Kaźmierczak, Alicja Bakalarz, Michał Wolny, Piotr Karwowski, Tomasz Stępień

**Affiliations:** Department of Mining, Faculty of Geoengineering, Mining and Geology, Wroclaw University of Science and Technology, Wybrzeże Wyspiańskiego 27, 50-370 Wrocław, Poland; magdalena.duchnowska@pwr.edu.pl (M.D.); urszula.kazmierczak@pwr.edu.pl (U.K.); alicja.bakalarz@pwr.edu.pl (A.B.); michal.wolny@pwr.edu.pl (M.W.); piotr.karwowski@pwr.edu.pl (P.K.); t.stepien@pwr.edu.pl (T.S.)

**Keywords:** crushed aggregates, grain composition, shape index, flakiness index, geometric properties of aggregates

## Abstract

The aim of this publication is to analyze the influence of rock mineral composition and rock geometric properties on the quality of crushed aggregates, from the perspective of selecting an adequate aggregate production technology. This research is based on samples of crushed aggregates from plants processing igneous rocks from four different igneous deposits. In the case of the geometric properties, shape and flakiness indexes were identified and subsequently analyzed along with particle size distribution. The performed tests allowed a conclusion that the shape of the particle is influenced by the mineral composition and size distribution. The grain size analysis demonstrated that flaky and non-cubical particles concentrate in the finest grain fractions, and the least variable shape index is observed for basalt aggregate. Some problems were also observed to exist in relation to the classification of grain shape. In the literature, the notions of regular and irregular grains seem to be used interchangeably with the notions of flaky and non-flaky grains. The performed tests show that flaky grains do not necessarily have to be non-cubical and vice versa. Therefore, this article proposes an approach in which the applied technique is precisely explained and the shape of grains is described with four notions: cubical, non-cubical, flaky, and non-flaky. The article also finally concludes that the next step in the research on selecting an optimal production technology of high-quality aggregates should be to analyze the selection of the fragmentation process while also characterizing the geometric properties of the aggregates.

## 1. Introduction

Mineral aggregates are the basic raw material used in housing and road construction [1,2,3,4,5,6]. In addition to mineral aggregate, other materials can be used in construction as substitute components for the production of building materials [7,8,9]. Nevertheless, a constant development of these two industries causes a heavy and still growing demand on rock raw materials. Currently, annual global consumption of aggregates already exceeds 40 billion Mg, 4 billion Mg of which is consumed in European Union. Ninety-one percent of the aggregates produced in European Union are obtained from natural deposits [10,11], and the production of crushed aggregates from high-quality rock raw materials accounts for almost 50% of the entire amount of aggregates used in Europe [12].

Natural crushed aggregates are obtained from compact rock deposits and are the most important rock raw materials for road and housing construction [13,14]. However, they have a variable quality, which depends on their structure and composition, and which may significantly influence the properties of a particular product. The produced crushed aggregates should nevertheless display adequate technical parameters, and thus—have high quality. Therefore, the production of such aggregates and the potential for their applications requires a detailed petrographic analysis and an investigation of the geometric properties of the rock material. Such an analysis should prove helpful in the selection of fragmentation process parameters and in mechanical classification, which is something of a novelty in approaching this problem. Therefore, the necessity for crushed aggregates to meet high requirements renders necessary the analysis of their structure and geometric properties. This fact is the motivation behind an attempt at such an analysis in order to point to problems related to the crushed stone aggregates. In addition, it is important to demonstrate the differences between the shape and flakiness index and to point out that these indices are not the same and cannot be used interchangeably. This may adversely affect the quality parameters of the produced aggregates. This approach will result in the identification of possible desired research possibilities to improve the quality of manufactured crushed stone aggregates.

## 2. Background

The properties of mineral aggregates are mainly defined on the basis of the lithology and mineral composition of the source rock material. Rock petrographic description may provide information on the probable characteristics of aggregates produced from rocks, but it cannot serve as the sole basis for evaluating aggregate properties [12,15]. This fact applies in particular to the geometric properties of aggregates, as the influence in this case is not only from petrography and mineralogy, but also from the production processes, and especially from the choice of technological processes involved in mechanical classification and fragmentation.

Crushed aggregate production is based on mining and mineral processing technologies, which involve three key processes: blasting, crushing in crushers, and mechanical classification. These processes are aimed at producing grains characterized by appropriate size and shape parameters as well as by physical and mechanical parameters, such as resistance to wear and fragmentation. At the stage of mining rocks for aggregate production purposes, the mined raw material is composed of coarse rock pieces, with a low content of the finest fractions [16]. The fragmentation and mechanical classification processes represent the most important stage of crushed aggregate production. The geometric parameters desired and expected by the client are achievable for any lithological type, on condition that the technological system is properly adjusted, in particular with respect to the number of fragmentation stages and the type of sieves used in the process of mechanical classification [17]. One of the main problems encountered during mechanical processing, transportation, and loading of the half-products and the finished products is the fact that some parts of the materials have a strong tendency for further fragmentation, which affects the geometric parameters of these materials [18].

The main geometric parameters include the size and shape of the particles and their structure, the latter one being mainly accounted for by the amount, size, and direction of fractures present in the tested material. Particle size distribution (PSD) of aggregates is an important parameter influencing a number of the properties of Portland cement concrete, such as workability, strength, durability, and volume stability [19,20,21]. Owing to a proper selection of the machine system, the obtained aggregates can be of high quality and have geometric properties desired by the client. Generally, the share of fine fractions and dusts in the product structure of the processing plant is assumed to be lower in open crushing systems than in closed crushing systems, which is a confirmation that the production technology has a significant influence on the geometric properties of the produced materials [22]. As a result of a multi-stage fragmentation process, the structure of the aggregate particles is frequently affected, as emphasized by Miskovsky et al. [23] and Gawenda [24]. Fractures that occur in the structures of individual aggregate particles on the one hand reduce the amount of energy needed to crush a particular fraction in the technological system, and on the other hand they reduce aggregate resistance to wear and fragmentation, thus lowering the strength parameters of the final product intended for use in the construction industry. The particle size, defined by the market demand, is a separate issue. Aggregates leaving the production process have different sizes, which advantageously allow them to be better adjusted to different purposes. Dust content significantly influences the aggregate quality. In general, their high content results in lower adhesion between the aggregate and the cement grout, significantly reducing the strength properties of concrete. However, Meisuh et al. [25] stress that the application of dusts in concrete may actually improve its strength properties.

The particle shape is another problem frequently observed in plants where aggregates are mechanically processed. The shape of aggregate particles can be described in terms of their form, angularity, and texture. Aggregate form can be understood as a description of its overall shape, in such terms as spherical, elliptical, rod-like, plate-like, etc. Angularity can be defined as the measure of sharpness of aggregate corners, for example, angular, sub-angular, round, etc. Aggregate texture is the measure of the smoothness of the aggregate surface [26]. Aggregate should be polyhedral and isometric, and it should have rough surfaces and sharp edges. Cubical shape also helps reduce the fracturing and crushing of aggregate particles. This phenomenon is of particular importance for aggregate used as ballast in railroads [27]. The shape of aggregate particles from a particular material is not identical for all of its fractions. In comparison to coarse grains, fine grains are significantly more elongated, have rough surfaces, and are more prone to wear and fragmentation along their fracture surfaces [28].

Particle shape is evaluated on the basis of current standards: EN 933-1, EN 933-3, and EN 933-4 [29,30,31]. Their scope includes identification of particle size distribution and particle shape of aggregates, as well as the content and quality of dusts. Particle shape is identified by determining the flakiness index (flaky and non-flaky particles) and the shape index (cubical and non-cubical particles). Other methods for identifying geometric parameters of aggregates involve 3D technologies, which are based on digital image analysis [32], photogrammetry [33], or laser scanning [27,34,35,36,37]. However, note should be made here that the use of the above technologies requires laboratories provided with expensive and complicated apparatus. In addition, such solutions are not standardized, leading to different interpretations of the results. For these reasons, standardized solutions seem more accurate and universal, allowing a comparison of the geometric properties of different aggregates.

In compacted asphalt mixtures, the spatial distribution and effective contact between stone particles depends on the shape, angularity, and surface texture of aggregate particles, especially for coarse aggregates [38]. Publications [27,39,40,41,42] confirm that particle shape has a significant influence on the mechanical properties and practical parameters of mineral–asphalt mixtures and railroad ballast. This issue was also investigated by Ostrowski et al. [43] who demonstrated that aggregate morphology has a significant impact on the strength and rheological parameters of different types of concrete. Liu et al. [44] confirmed that the use of spherical and angular aggregates in a stone matrix asphalt may improve the resistance of the mixture to permanent deformation, and the use of more spherical and less flaky aggregates improved the fatigue performance. Also, Stempkowska et al. [45] confirmed in their research that the arrangement of aggregate particles in the material and their shape are of major importance for the mechanical strength of concrete. Concrete mixtures with a 100% content of regular particles proved to have an approximately 10% greater compressive strength than other samples with irregular particles. The above observations allow a conclusion that the higher the content of flaky and longitudinal particles, the lower the resistance to fragmentation. Thus, knowledge of aggregate morphological characteristics, including shape and flakiness index, is essential for quality control of aggregate production [46].

In conclusion, the quality and the geometric properties of aggregates produced from rocks depend on the lithological and mineral characteristics of the processed material, on the adequate selection of the machines used in the fragmentation and classification of aggregate [47], as well as on the applied technological process, which translates into the number of fragmentation stages and the balance of mass flow over a particular time period [13].

## 3. Materials and Methods

### 3.1. Materials

**Figure 1:**
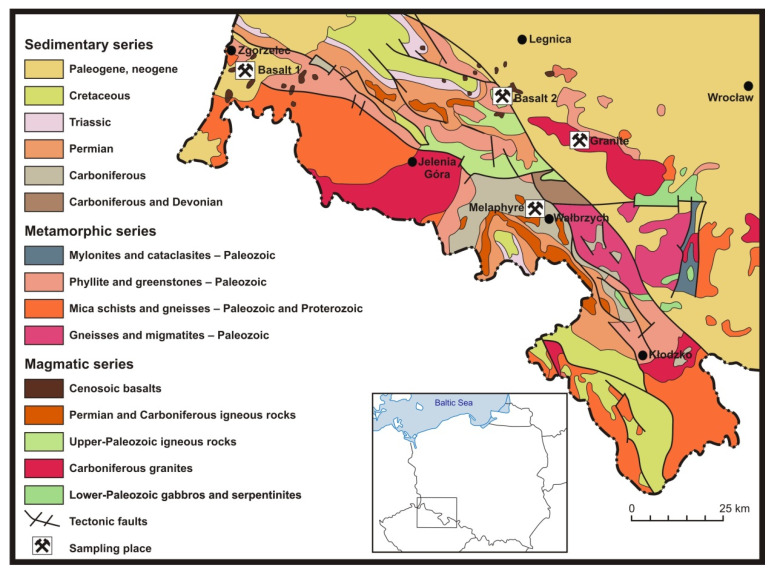
Locations in which the specimens were collected, shown on the geological map of the Lower Silesia region (own work based on [48]).

The material for the laboratory tests here presented consisted of specimens of crushed aggregates from plants processing igneous rock raw materials from four different deposits. Four averaged specimens of crushed aggregates, each having a minimum mass of 120 kg, were collected for the tests. Their particle sizes were 0–31.5 mm. The locations where the specimens were collected are shown on the geological map of the Lower Silesia region (Figure 1).

**Figure 2:**
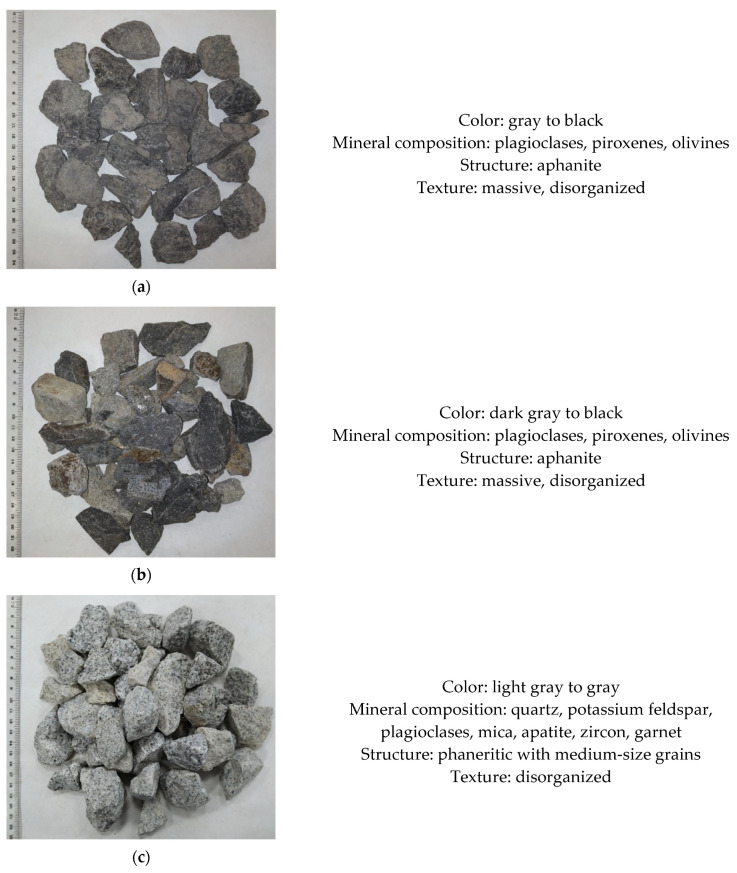

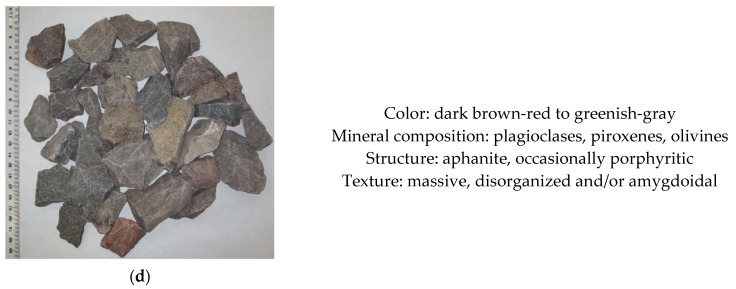
Petrographic characteristics of the investigated aggregates from igneous rocks: (**a**)—BASALT 1; (**b**)—BASALT 2; (**c**)—GRANITE; (**d**)—MELAPHYRE.

The collected specimens included two types of basalt aggregates. A note should be made that from the petrograhic perspective, basalt 1 from the Lubań region is a Cenozoic igneous eruptive rock formed from nephelinite and tephrite lavas, in which aphanite background comprises more than 80% of the rock, and contains phenocrysts and microphenocrysts of olivine and clinopiroxene, as well as magnetite. Basalt 2 was collected from a deposit in the vicinity of Złotoryja, located approx. 50 km in a straight line from the basalt 1 deposit. As in the case of the first specimen, its aphanite background comprises phenocrysts of piroxene and olivine, and occasionally also air bubbles filled with secondary carbonate minerals. The investigated basalt aggregate also contains particles of volcanic tuffs. The granite aggregate specimen was obtained from the processing of Carboniferous granites from the Strzegom region. This granite has a phaneritic structure with medium-size grains, and it contains feldspar, quartz, and mica minerals. The last specimen was melaphyre collected from a deposit in the Wałbrzych region. It is an alkaline eruptive rock having a porphyritic structure and amygdoidal texture. Its composition is dominated by fine plates of intensively interpenetrating plagioclases, piroxenes, olivines, and silica from secondary crystallization. Petrographic description of the analyzed aggregates is shown in Figure 2.

The specimens were collected from the technological lines of aggregate processing plants. After being subjected to preliminary fragmentation with the use of blasting materials, the rocks were transported to the processing plants on haul trucks. Subsequently, they passed preliminary selection, preceded by crushing oversize blocks in a conical crusher—this was the pre-crushing stage in gyrators. After it was pre-crushed, the material was passed to the first crushing stage in a jaw crusher (in the case of basalt aggregate) and in conical crushers (in the case of granite and melaphyre aggregate). The initial capacity of the process systems was less than 400 Mg/h. The material collected for tests passed only the first crushing stage, and the particles sized 0–31.5 mm were separated during the mechanical classification process in vibrating sieves.

### 3.2. Methods

The test methodology was based on our own observations and on European standards. Our own observations served to prepare a petrographic analysis of the material, while the geometric properties (particle size distribution, particle shape based on the flakiness index and the shape index) were identified on the basis of the current standards EN 933-1, EN 933-3, and EN 933-4 [29,30,31].

The structure of the mineral aggregates was evaluated under a microscope, identifying the roughness and edge sharpness of the particles. Additionally, the presence of fractures was verified with the use of a Motic SMZ-168 Series microscope (Motic, Hong Kong, China), a Nikon Digital Sight DS-Fi2 camera (Nikon, Tokyo, Japan) and Nikon NIS-Elements Advanced Research software (ver. 3.00). The above features were identified for a representative sample consisting of aggregates 8–10 mm and 16–20 mm in size. Observations were made for all aggregate particles in the samples. Then, about 20% by mass of particles from each samples were selected to take a picture. The most representative particles were selected and presented in figures.

Identification of particle size distribution (as per EN 933-1) consists in separating the material into fractions with the use of square sieves having the following aperture sizes: 0.063, 0.125, 0.25, 0.5, 1.0, 2.0, 4.0, 5.6, 6.3, 8.0, 10.0, 11.2, 12.5, 14.0, 16.0, 20.0, 22.4, and 31.5 mm. The minimum mass of the test specimen was 10 kg. The tests were performed in wet conditions in order to increase the effectiveness of the classification process. It also allowed a precise separation of the dust fraction from the tested aggregate. After the classification process, the specimens were dried for 24 h in a laboratory drier, at 105 °C. When dry, they served to identify mass expenditures for each particle size class, which in turn were used to prepare PSD curves.

The flakiness index (EN 933-3) and the shape index (EN 933-4) were identified for the aggregate already divided into separate fractions in the above process. The mass of the test specimen was 10 kg. The grain flakiness index is identified in two sieving stages. The first stage includes the same range as in the case of the PSD identification. In the second stage, the aggregate is sieved on bar sieves, which have parallel slots D/2 in width. The flakiness index for individual fractions is calculated as the mass of particles that pass the bar sieves, and is expressed as a percentage in relationship to the mass of the particles of the particular size fraction. As a result, flaky particles (which pass through the bar sieves) are separated from non-flaky particles (which remain on the bar sieve). The shape index of aggregate particles is identified on the basis of the ratio between the maximum dimension of the grain, defined by the greatest distance separating two parallel planes tangential to the particle surface (L), and the thickness of the minimum grain dimension, defined by the smallest distance separating two parallel surfaces tangential to the particle surface (E). The test is performed with the use of a particle slide gauge. The particle shape index is calculated as a mass of grains having a ratio between the L/E dimensions greater than three, and is expressed as a percentage of the total mass of the tested particles. When the dimensional ratio L/E is greater than 3, the particle is classified as cubical. Otherwise the particle is classified as non-cubical.

## 4. Results and Discussion

**Figure 3:**
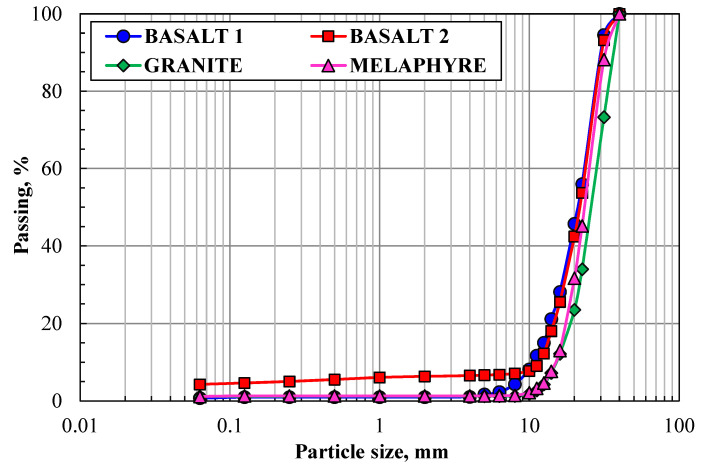
Particle size distribution of aggregates.

The most important parameter describing the geometric properties of aggregates is their particle size distribution. PSD classification is performed on the basis of a sieving operation, which is intended to divide a specimen of aggregate into fractions consisting of particles having specific size limits. Figure 3 shows the results of PSD classification for the tested aggregates. The basalt aggregates have similar particle sizes, except for the finest fractions. The basalt 1 aggregate has a higher content of fine particles, which may indicate that it is less resistant to fragmentation processes. The granite and melaphyre aggregate showed similar characteristics of the fine fraction curve, albeit above 15 mm the grains of granite were coarser.

The content of fine particles, which are decisive for the quality of the produced aggregate, is an important parameter of this construction material. Table 1 shows the results of PSD classification for fine grains, below 0.063 mm. The basalt 2 aggregate has the highest content of this grain size, which can be attributed to the fact that it seemed more prone to fragmentation already under the macroscopic evaluation, as it revealed high tuff content.

**Figure 4:**
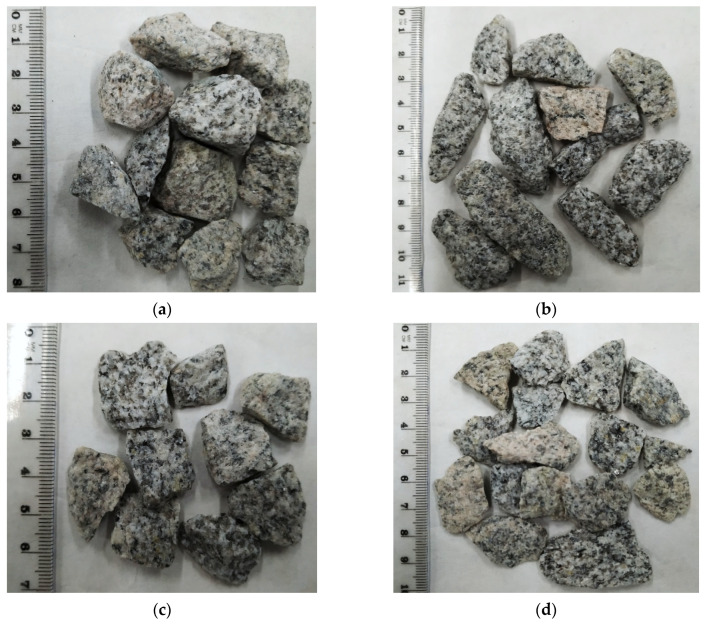
Geometric characteristics of aggregate particles for granite (size fraction 14–16 mm): (**a**)—non-flaky cubical particles; (**b**)—non-flaky non-cubical particles; (**c**)—flaky cubical particles; (**d**)—flaky non-cubical particles.

Wang et al. [38] and Heidelberg et al. [49] highlight that different definitions of shape, angularity, and texture are used, and the results of these morphological characterizations are not comparable with each other. In the literature, aggregate particles are classified as either regular or irregular, often meaning flaky or non-flaky [43,45]. European test methods of aggregate geometric parameters do not mention such classification, and therefore such an approach may be confusing, as flaky particles do not have to be necessarily non-cubical and vice-versa. It is thus important to stress what authors mean when they use the notions of ‘regular’ and ‘irregular’ in relation to the commonly accepted test methods. Also, it seems more effective to define aggregate particles in accordance with the standards, indicating whether the particles are flaky, non-flaky, cubical, or non-cubical. Figure 4 shows an example of granite particle classification with respect to the shape and flakiness indexes—particle grade of 14–16 mm. The flakiness and shape indexes were identified in a standardized procedure for averaged aggregate samples, and each measurement was repeated five times so as to eliminate the measuring error. The values of the obtained indexes were different by no more than 1.5%. Table 2 shows the averaged results of the flakiness and shape indexes for the analyzed size fractions of the aggregates.

The analysis of the data from Table 2 indicates that the finer the size fraction, the higher the content of non-cubical grains, and thus the higher the value of the shape index for all of the analyzed igneous aggregates. The case is similar for the flakiness index, where non-flaky particles dominate except for the phaneritic granite aggregate, in which the flakiness index is very low for the coarsest particles.

**Figure 5:**
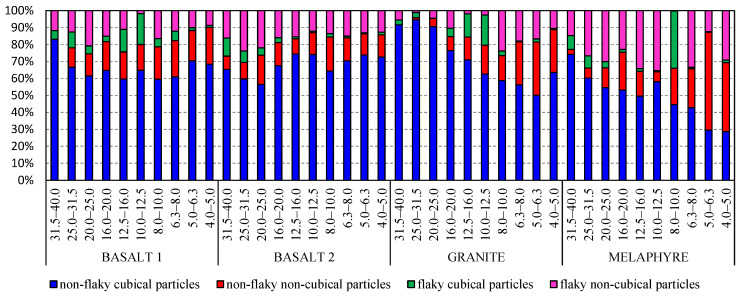
Analysis of shape and flakiness indexes for different size fractions.

**Figure 6:**
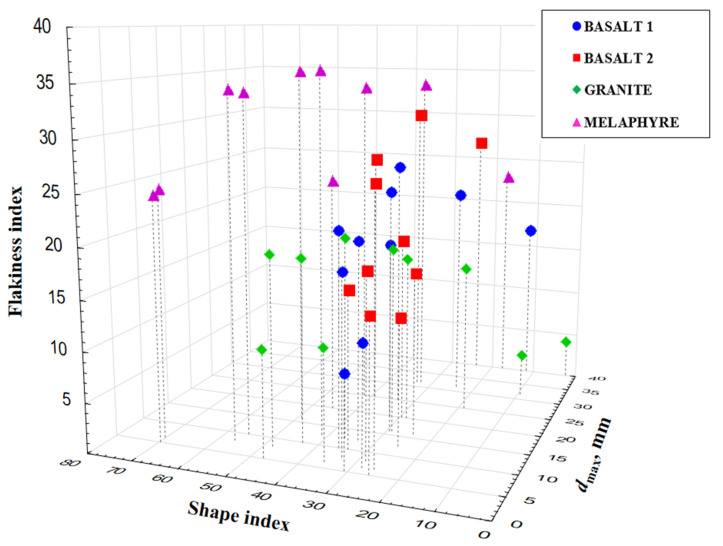
Comparison of shape and flakiness indexes for different size fractions.

The tests clearly indicated that not all of the analyzed cubical particles will be classified as non-flaky particles and vice-versa; the group of definitely flaky particles may contain cubical particles, which is a fact that is not pointed to at all in the literature. This observation is confirmed by the results presented in Table 2 and Figure 5. Each of the analyzed fractions includes flaky, non-flaky, cubical, and non-cubical particles. Interestingly, they represent a certain proportion of the total feed. Figure 6 is a three-dimensional graph showing the relationship between the size distribution and the values of the shape and flakiness indexes. The spatial distribution of the results allows an observation that the least variable content of non-cubical particles is found in the basalt aggregate for all of the particle size grades. Also, the coarser the mineral particles, the higher the content of cubical particles in the specimen. Similarly, based on the literature, particles with a grain size of less than 2 mm [28] are much more elongated and/or flaky than large particles. However, some works encounter assumptions that the shape index does not depend on the particle size [50,51]. The relationship is different for the flakiness index of the basalt aggregate. The content of flaky particles was observed to significantly increase with an increase in the coarseness of the aggregate, while the shape index is within a narrow range of 20–40% relative to the granite and melaphyre aggregates. The spatial structure of the point distribution for the basalt aggregate was observed to be at a similar level, and the higher content of non-cubical particles in the basalt 2 aggregate may be a result of its lower resistance to mechanical impacts due to high content of basalt detritus and volcanic tuffs. The analysis demonstrated that cubical non-flaky particles dominate in the tested aggregates and that in the case of the granite and melaphyre aggregates, the content of non-flaky cubical particles decreases in the finer size fractions. At the same time, the content of non-flaky but also non-cubical particles increases. In comparison, Xing et al. [47] shows that the distribution of the contour shape (or angularity) parameters of mineral fillers prepared using the same grinding process and sieving method varied according to mineralogical composition and particle size. Most of the particles had similar morphological characteristics in terms of outline shape (or angularity).

The above tests indicate that in order to be more standardized, the terminology applied to aggregates should include their geometric properties: flaky, non-flaky, cubical, and non-cubical. Their importance is well represented in the test methodology of the shape and flakiness indexes for mineral aggregates.

**Figure 7:**
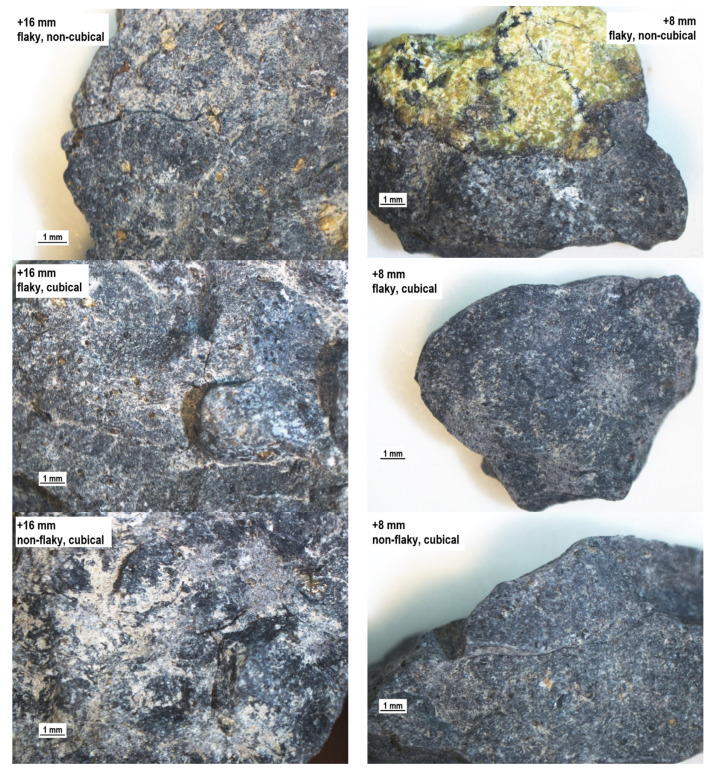

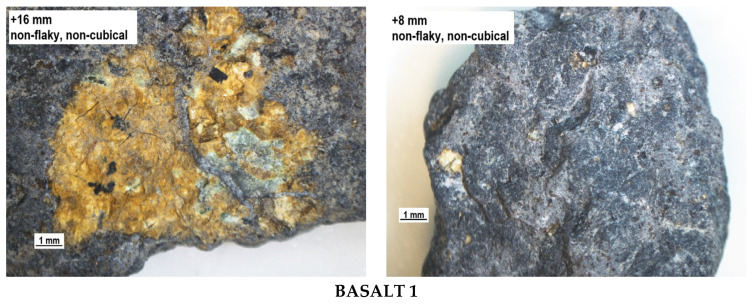
The structure of the particles of the basalt 1 aggregate, separate for the shape and particle size.

**Figure 8:**
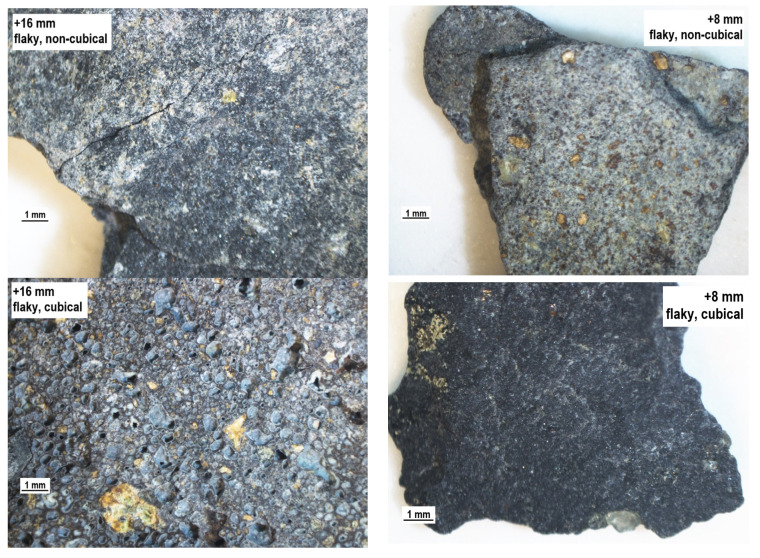

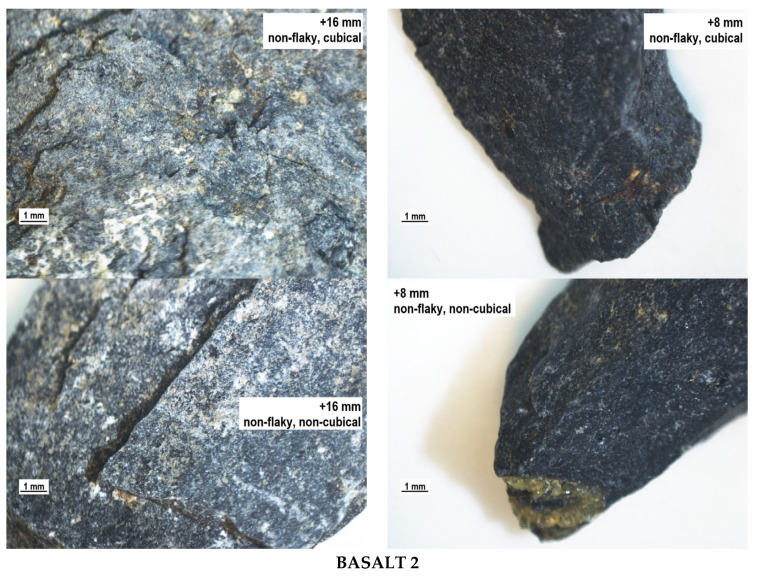
The structure of the particles of the basalt 2 aggregate, separate for the shape and particle size.

**Figure 9:**
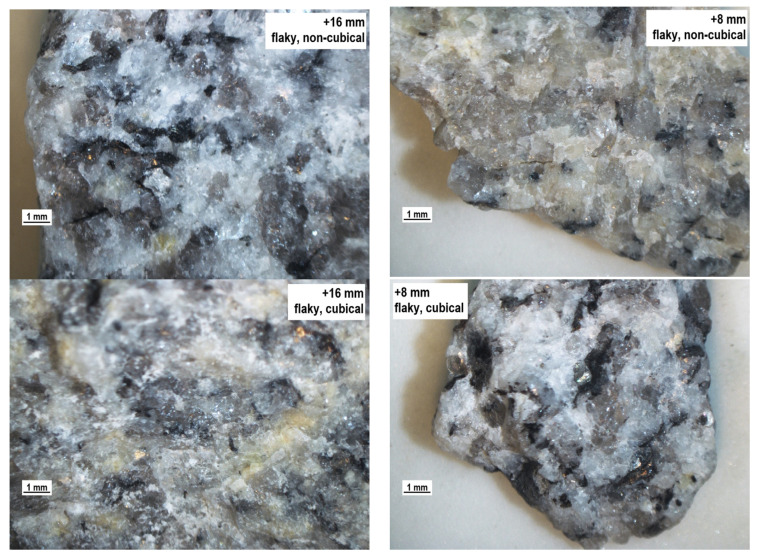

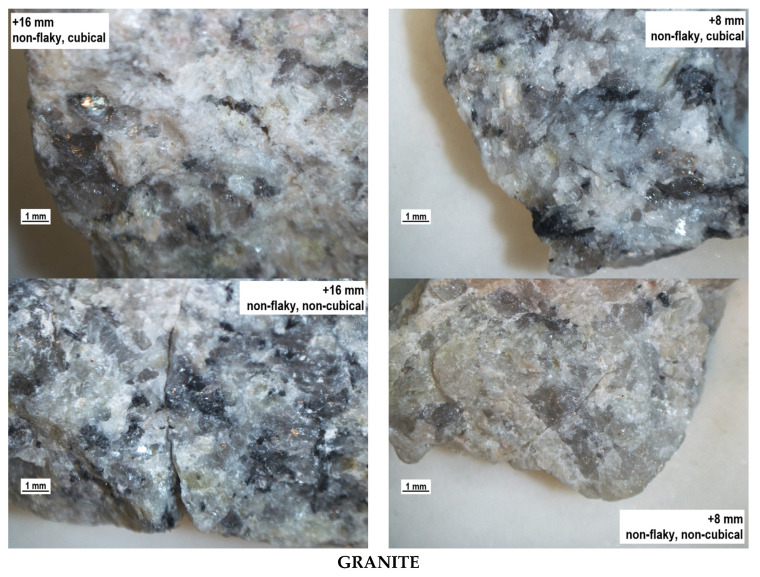
The structure of the particles of the granite aggregate, separate for the shape and particle size.

**Figure 10:**
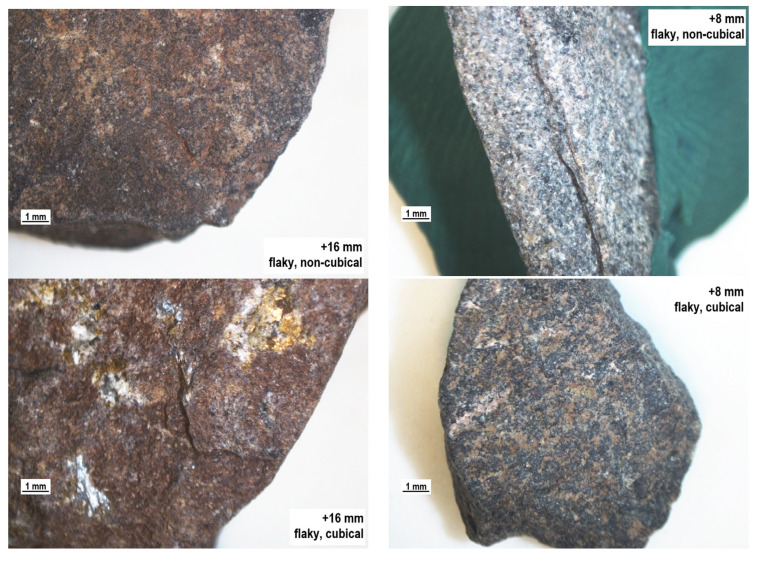

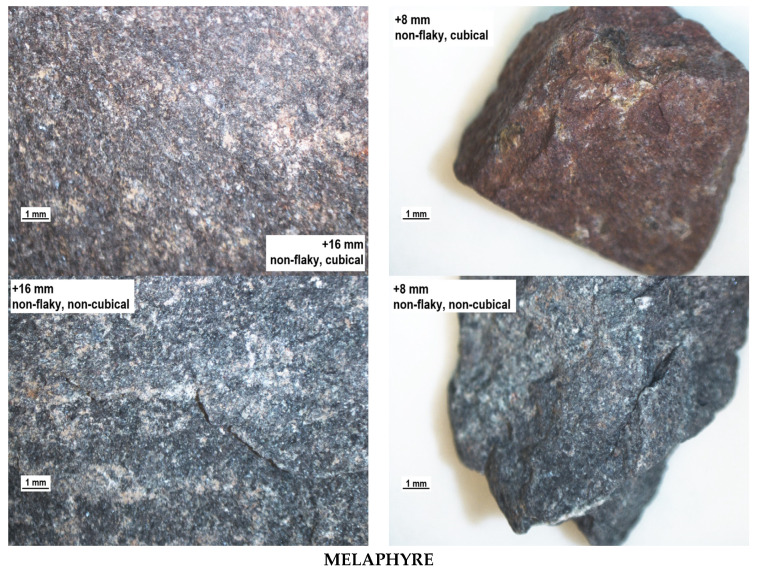
The structure of the particles of the melaphyre aggregate, separate for the shape and particle size.

The production processes providing aggregates with expected geometric properties invasively modify the rock structure, leading to numerous fractures. This phenomenon has a negative influence on the process of evaluating aggregate quality, as particle strength is affected, leading to reduced durability. Therefore, it seems justified to supplement the evaluation of the geometric parameters of aggregates with the evaluation of the structure of mineral particles. Figure 7, Figure 8, Figure 9 and Figure 10 show a microscope analysis of the structure of mineral particles with allowance for the shape and flakiness indexes and for the particle size fraction. Two fractions were used in the analysis: +16 mm and +8 mm. The particles are shown in the following order: flaky non-cubical particles, flaky cubical particles, non-flaky cubical particles, and non-flaky non-cubical particles, for the +16 mm fraction (on the left) and for the +8 mm fraction (on the right), respectively. It should be pointed out that fractures were observed in about 20–30% of particles in the tested samples.

In the case of the coarser fraction of basalt 1 aggregate, the fracture line structure is not related to the arrangement of phenocrysts. Additionally, the fractures were observed to occur mainly from the external boundary of the particle and extend centrally towards the center. These are characteristic of flaky non-cubical particles. For the remaining fractions, fractures are rare. It was also observed that in the case of coarser fractions, the majority of tuff particles are classified as cubical non-angular particles. In the case of finer fractions (below 8 mm), the main fractures are found on the boundary of the aphanite background and phenocrysts (Figure 7).

Similar as in the case of the basalt 2 aggregate (Figure 8) for the +16 mm fraction, fractures occur more frequently in the non-cubical particles than in the cubical particles. In addition, structures of internal fracture lines are clearly visible in the phenocrysts. In the case of fine particles (below 16 mm), fractures are found mainly in the structure of the phenocrysts or on the boundary between the phenocrysts and the background. Fractures are rarely observed in cubical particles.

The granite aggregate (Figure 9) has a low content of fractures in both of the analyzed size fractions. The fractures were mainly related to the structure of the mica minerals. The flaky thin particles were observed to be more prone to fractures.

The granite aggregate has a massive structure and the fractures in their structure were observed rarely, only in the case of thin particles. Unlike in the basalt aggregate, fractures in the granite material did not occur on the boundary between the background and the crystals visible against it, such as secondary mica particles (Figure 10).

The analysis of fractures present in the particles with allowance for the shape and flakiness indexes can be summarized by a conclusion that, in the case of the analyzed igneous aggregates, fractures occur more frequently with the increasing particle flakiness. Additionally, in the case of the basalt aggregates, the structure of the fractures is strongly correlated with the boundary of the aphanite background and the phenocrysts. The analysis of the structure of the fracture lines and their directions indicates that the fractures extend due to stresses that occur during the crushing process (the applied fragmentation technology). In addition, the number of fractures is greater in fine flaky particles than in coarse flaky particles.

## 5. Conclusions

Crushed aggregates must meet high quality-related requirements, and their variable quality due to the type of rock may thus be a negative factor. Therefore, the selection of a proper technology for producing crushed aggregates should be in the first place preceded by a detailed petrographic analysis of the rock material, performed in connection with the evaluation of its geometric properties.

The research demonstrated that the particle shape is influenced by the petrographic properties, but also, to a significant extent, by the particle size, which is the result of the applied fragmentation technology. The tests demonstrated that flaky particles and non-cubical particles concentrate in the finest grain fractions. However, the content of non-cubical particles is similar for all of the analyzed size grades. The analysis of the geometric properties also demonstrated that the content of fine particles in the test specimens was comparable for granite and melaphyre. The basalt aggregates, on the other hand, provided the two extreme results, which can be attributed to the presence of tuffs. Additional problems were related to the classification of the shape of mineral particles. The tests and the analysis of their results demonstrated that flaky particles dominate in the finer grain fractions. This fact is of importance, as the lower the content of flaky particles, the higher the resistance to fragmentation. The tests also demonstrated that the basalt aggregate has the highest content of non-cubical particles in relation to the granite and melaphyre aggregates.

At this point, it is worth noting that in the literature the terms of regular and irregular particles are frequently used instead of the terms of non-flaky and flaky particles. The authors believe this interpretation to be incorrect. Flaky particles do not have to be non-cubical and vice-versa. Therefore, the distinction between the four notions is very important for the classification of crushed aggregates. Therefore, a classification of particles as regular or irregular should be accompanied by a precise explanation of the applied measurement technique in order to avoid confusion about the conclusions of such analyses.

The tests also demonstrated that the particle shape has a major influence, along the petrographic properties, on the particle size distribution, which is the result of the applied fragmentation technology. Flaky particles and non-cubical particles concentrate in the finest grain fractions. In addition, the structure of such particles more frequently includes fresh fractures, which is of importance and is related to the lowered resistance to wear and fragmentation. The tests also demonstrated that fine flaky particles have more fractures than coarse particles have, and the fractures are the result of the applied fragmentation technology. Therefore, an analysis of the influence of the fragmentation technology on the quality parameters of aggregates should be further investigated and will be the next object of focus in this research.

Therefore, considering the conclusions of the conducted research, it can be concluded that further research stages that focus on the quality of produced crushed aggregates should be:

- an analysis of the influence of the fragmentation technology on the quality parameters of aggregates,
- an analysis of parameters for the selection of mechanical classification of crushed aggregates, and
- development of a methodology for selection of a technology system for production of crushed aggregates (including lithology and mineralogy of the material).

## Figures and Tables

**Table 1:** Fine particle content (in %) in the tested aggregates (particle grade below 0.063 mm).

BASALT 1	BASALT 2	GRANITE	MELAPHYRE
0.69	4.26	1.12	1.13

**Table 2:** Average shape and flakiness indexes for the analyzed igneous aggregates.

Particle Size, mm	*d*_max_, mm	BASALT 1		BASALT 2		GRANITE		MELAPHYRE	
		Shape Index	Flakiness Index	Shape Index	Flakiness Index	Shape Index	Flakiness Index	Shape Index	Flakiness Index
31.5–40.0	40.0	11.77	16.87	23.94	26.84	2.43	4.12	17.50	22.92
25.0–31.5	31.5	24.06	21.96	33.59	30.56	9.32	4.57	32.54	33.84
20.0–25.0	25.0	33.84	25.51	39.15	26.26	18.72	15.30	41.74	33.76
16.0–20.0	20.0	32.21	18.27	29.56	18.82	28.46	16.91	45.23	24.52
12.5–16.0	16.0	29.33	24.32	24.37	16.60	28.90	18.62	48.92	35.69
10.0–12.5	12.5	33.53	20.03	25.10	12.97	36.36	20.22	41.40	36.01
8.0–10.0	10.0	35.62	21.36	33.68	15.86	43.41	18.44	55.30	33.96
6.3–8.0	8.0	33.42	18.04	28.51	18.34	48.03	19.01	56.33	34.32
5.0–6.3	6.3	27.98	12.01	25.62	26.72	35.72	11.11	70.22	24.82
4.0–5.0	5.0	30.58	9.33	25.69	15.05	47.17	10.73	69.78	24.55

## Data Availability

Not applicable.
